# Annotations

**Published:** 1904-01-30

**Authors:** 


### ANNOTATIONS.

### Pigmentation in Addison's Disease.

THERE is much that is obscure in the phenomena of Addison's disease, and in many respects indeed it is not at present possible to advance beyond the limits of mere empirical observation. Thus it is difficult to suggest the relationship between excessive pigmentation of the skin on the one hand and disease of the suprarenals and neighbouring nervous structures on the other. The broad fact, however, remains that there is some such relationship. Again, it is certain that the adrenals may be extensively diseased without pigmentary changes in the integuments, and equally there are frequent instances of such changes as melanoderma and leucoderma apart from appreciable alteration in the structure of the suprarenal bodies. An interesting point in connection with this subject is the question whether in Addison's disease the pigmentary disturbances are always in the direction of excess or whether, on the other hand, the more or less widespread darkening of the surface is ever interrupted by unduly pale areas (leucoderma). A reference to Addison's original essay shows that among his cases he figures one in which the leucodermic pattern of pigmentation was conspicuous and though no post-mortem examination was obtained Addison himself had no doubt of the accuracy of the diagnosis. Further he records another instance in which such an examination was made and in which the characteristic changes in the suprarenals were present. An attempt has been made to challenge or explain away these records and with so large a measure of apparent success that the leading manuals of medicine announce almost without qualification that in Addison's disease the pigmentation of the skin is more or less generally increased. This was certainly not Addison's teaching, and further some later observers have described experiences which confirm the view that the presence of pale areas of skin in the midst of the general darkening does occur in occasional instances of the disease which bears Addison's name. When it is said that these observations rest on the authority of such competent observers as Gairdner, McCall Anderson, and Byrom Bramwell, it must be allowed that leucoderma though often due to other causes may be a consequence and index of disease of the suprarenal bodies.

### Amusements for the Blind.

OUR pity for the afflicted is apt to take too pathetic a form, and to make us forget that our brethren who lack limbs, or sight, or hearing, are creatures of like natures with ourselves, and require, besides being educated, and improved, and sympathised with, to be occasionally amused. A visit to the Royal College of Music for the Blind at Norwood will prove to any one how much fun survives among these afflicted ones, and their rowing, swinging, cycling, and stilt-walking will show that their lack of sight does not render even fairly active exercise impossible for them. But in many homes, and in the winter evenings in all, what amusement is provided for a member of the family who happens to be blind? Books in Braille type are expensive, so perhaps one is not justified in grumbling that those available often include only educational works. How many people think of providing blind friends with less intellectual recreations? Yet it is worth while to remember that the blind can play chess or draughts, or cards. There are prepared boards, with squares alternately plain and chequered, and with holes in the centre of each wherein to fix the pieces, which make it possible for the blind to play either chess or draughts as easily, or nearly so, as ordinary mortals. Then there are cards, with the ordinary symbols for sighted partners, but with little raised dots at the margin to explain to the sightless the value of each bit of pasteboard. With these the blind can join in a game of whist as well as their neighbours. For younger people there are the quartette games which delighted our childhood, with differing combinations of dots at the margin of the cards to show the blind player which represents Master Dose, the doctor's son, and which Miss Bung, the brewer's daughter. In such a matter the humbler task may be the greater. Since all blind people are not, by mere virtue of their misfortune, superior to their sighted neighbours it is the truest kindness to bring them into touch with people of their own age and capacity, and if they can join in the ordinary amusements of life with those who see, it will help greatly to make them, as far as may be, normal.

### The Noble Art of National Defence.

MR. RUDYARD KIPLING has put in a plea for a form of conscription which should appeal to all parents of public school boys. He points out that, while British public sentiment strongly objects to conscription for the army, a form of conscription exists in schools with regard to games. A boy must play cricket in summer and football or hockey in winter, whether he likes it or not, and masters and parents alike are agreed that this kind of compulsion is good. The wholesome and hardening effect of these games is undeniable; the only point in question is whether these same effects might not be attained by the substitution for them of a military training which would, in addition, make the boys fit, if need arose, to take their part in defending their country with more knowledge of the work than they have at present. Most of our schools have indeed cadet corps, but the joining of these is optional, and even parents who would, on principle, gladly see their boys join one, are kept back by the doubt lest the addition of drill to games may interfere too much with that intellectual training for which, after all, the school is supposed to be chiefly intended. The circular issued by one public school says: "Drills and shooting practice have been arranged so as not to interfere with a boy's cricket or football games." But drills and shooting, being physical training of a very useful kind, might, in most cases, take the place of these games. The boy who leaves his school a good shot, able to bear the fatigues of an ordinary field day, and to stand the rough and tumble of camp life will not be a weakling. Not only will he be strong, but he will be alert, ready to obey the word of command, able to act in concert with his fellows. He will have learned already the first steps of soldiering, and if his country's need should call upon him, he would be of more use in the field than a lad who had all this to learn.

